# Alternative splicing and differential subcellular localization of the rat FGF antisense gene product

**DOI:** 10.1186/1471-2199-9-10

**Published:** 2008-01-23

**Authors:** Shuo Cheng Zhang, Kimberley A MacDonald, Mark Baguma-Nibasheka, Laurette Geldenhuys, Alan G Casson, Paul R Murphy

**Affiliations:** 1 Department of Physiology and Biophysics, Dalhousie University, Halifax, Nova Scotia B3H 1X5, Canada; 2 Department of Pathology, Dalhousie University, Halifax, Nova Scotia B3H 1X5, Canada; 3 Department of Surgery, Dalhousie University, Halifax, Nova Scotia B3H 1X5, Canada; 4 Department of Surgery, University of Saskatchewan, Royal University Hospital, Saskatoon, Saskatchewan S7N 0W8, Canada

## Abstract

**Background:**

GFG/NUDT is a nudix hydrolase originally identified as the product of the fibroblast growth factor-2 antisense (FGF-AS) gene. While the FGF-AS RNA has been implicated as an antisense regulator of FGF-2 expression, the expression and function of the encoded GFG protein is largely unknown. Alternative splicing of the primary FGF-AS mRNA transcript predicts multiple GFG isoforms in many species including rat. In the present study we focused on elucidating the expression and subcellular distribution of alternatively spliced rat GFG isoforms.

**Results:**

RT-PCR and immunohistochemistry revealed tissue-specific GFG mRNA isoform expression and subcellular distribution of GFG immunoreactivity in cytoplasm and nuclei of a wide range of normal rat tissues. FGF-2 and GFG immunoreactivity were co-localized in some, but not all, tissues examined. Computational analysis identified a mitochondrial targeting sequence (MTS) in the N-terminus of three previously described rGFG isoforms. Confocal laser scanning microscopy and subcellular fractionation analysis revealed that all rGFG isoforms bearing the MTS were specifically targeted to mitochondria whereas isoforms and deletion mutants lacking the MTS were localized in the cytoplasm and nucleus. Mutation and deletion analysis confirmed that the predicted MTS was necessary and sufficient for mitochondrial compartmentalization.

**Conclusion:**

Previous findings strongly support a role for the FGF antisense RNA as a regulator of FGF2 expression. The present study demonstrates that the antisense RNA itself is translated, and that protein isoforms resulting form alternative RNA splicing are sorted to different subcellular compartments. FGF-2 and its antisense protein are co-expressed in many tissues and in some cases in the same cells. The strong conservation of sequence and genomic organization across animal species suggests important functional significance to the physical association of these transcript pairs.

## Background

**Figure 1:**
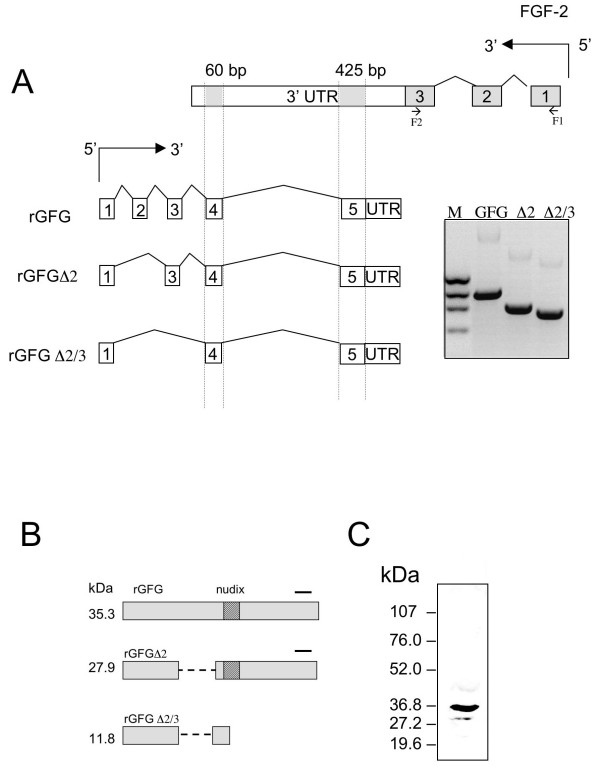
**Alternative splice variants of the FGF antisense gene and encoded *GFG *protein isoforms**. (A) Alternative mRNA splicing of GFG mRNA transcripts. The dashed lines indicate the regions of complementarity with exon 3 of the FGF2 gene on the opposite DNA strand. *Inset*: RT-PCR detection of alternative splice variants. (B) The predicted translation products of the alternatively spliced GFG mRNAs, aligned against the common nudix motif. The black line indicates the region detected by the anti-GFG antiserum (C) Western blot detection of 35 kDa and 28 kDa GFG isoforms in rat liver homogenate.

The FGF-2 gene is post-transcriptionally regulated by an endogenous complementary (antisense) mRNA transcribed from the GFG/NUDT6 gene on the opposite DNA strand (Fig. 1). The FGF antisense (FGF-AS) transcript was first identified in *Xenopus laevis *[1] and has since been identified in a variety of other vertebrate species including chicken [2], rat [3] and human [4,5]. The highly conserved organization and sequence of the FGF-2 and GFG genes across many vertebrate species suggest that this structural relationship has an important function. The sense and antisense RNAs form stable dsRNA complexes *in vivo *and it is believed that antisense RNA plays a role in the regulation of FGF-2 mRNA stability [1] and translation [6]. The inverse association between FGF-2 and FGF-AS mRNA levels in a variety of tissues during development supports the notion of a regulatory function of the antisense RNA [2,4,7,8]. We recently reported that overexpression of FGF-AS reduced cellular FGF-2 content and delayed S-phase progression in a rat glioma cell line [9].

In addition to its role as a regulatory RNA, the FGF-AS transcript encodes GFG, an evolutionarily conserved nudix motif protein of unknown function [10]. GFG belongs to the Nudix hydrolase (NUDT) superfamily, characterized by a consensus signature sequence GX_5_EX_7_REUXEEXGU (the Nudix box), where X may be any amino acid and U represents one of the bulky hydrophobic amino acids, usually Ile, Leu or Val [11,12]. The major substrates of these hydrolase enzymes are *nu*cleoside *di*phosphates linked to some other moiety *X*, hence the acronym Nudix [11]. The founding member of this family, the prokaryotic MutT protein, is responsible for removing 8-oxo-dGTP from the nucleotide pool, thus preventing transversion mutations caused by mis-incorporation of 8-oxo-guanine residues into DNA [13]. The InterPro database [14] currently contains 2226 nudix motif proteins from over 360 species ranging from viruses to man. Nudix hydrolases can be grouped into distinct subfamilies according to their specificities for substrates, including intact and oxidatively damaged (deoxy)nucleoside triphosphates, dinucleoside polyphosphates, nucleotide sugars, NADH, ADP-ribose, dinucleotide coenzymes, and mRNA (reviewed in [11,15]).

In humans, alternative splicing of the FGF-AS mRNA gives rise to 3 isoforms of GFG, the largest of which contains a mitochondrial targeting sequence (MTS). We recently demonstrated that the MTS is necessary and sufficient for mitochondrial targeting of hGFGa, whereas hGFGb and hGFGc are localized in the cytoplasm and nucleus [16]. In rat the FGF-AS mRNA is also alternatively spliced, resulting in at least 3 transcripts [6], but the subcellular distribution of their protein products is unclear. Our initial immunohistochemical characterization using antibodies against the nudix domain, and C-terminal peptide of rat GFG, indicated a predominantly nuclear localization in liver. However, Western blotting of liver subcellular fractions identified GFG immunoreactivity in both the nuclear and mitochondrial fractions [17]. In contrast, localization was exclusively cytoplasmic in transiently and stably transfected rat pituitary-derived GH4 mammosomatotroph cells [18] but both cytoplasmic and nuclear in a rat lymphoma cell line [19]. The diverse subcellular localization of GFG could reflect either differential subcellular trafficking of the alternatively spliced isoforms, or interaction with lineage-dependent cell-specific factors. Determining the subcellular localization of GFG isoforms may provide insight into their function in the cell.

We focused here on elucidating the expression and subcellular distribution of the previously identified rat GFG isoforms [6]. Using computational analysis, we identified a putative mitochondrial targeting signal peptide (MTS) at the N-termini of GFG proteins in a variety of animal species including rat. Confocal laser scanning microscopy of cells transiently or stably transfected with individual GFG-GFP fusion constructs and deletion mutants revealed that recombinant rat GFG isoforms are targeted to mitochondria by means of the N-terminal MTS, while GFG proteins lacking the MTS are distributed in cytoplasm and nucleus. This pattern of distribution is consistent with the tissue specific expression of GFG in a variety of tissues.

## Results

### Computational analysis of GFG mRNAs and their protein products

The rat *FGF2/GFG *gene locus maps to 2q25 and covers more than 34 kb. The full-length rat FGF-2 mRNA contains 3 exons and encodes at least 4 FGF-2 isoforms derived by alternative translation initiation. The overlapping GFG mRNA contains five exons and encodes a 35 kDa nudix-motif protein [3]. Two alternative splicing variants, *GFGΔ2 *(supporting clone, AAF07934) lacking exon 2 and *GFGΔ2/3 *(AAF07935) lacking exons 2 and 3 encode, respectively, rGFGΔ2, a 28 kDa nudix-motif protein, and rGFGΔ2/3, a 12 kDa protein lacking a nudix motif [6]. Antisera against the COOH-terminal or the Nudix domain detect the full length (35 kDa) and *GFGΔ2 *(28 kDa) *isoforms *in homogenates of rat C6 glioma cells and in normal rat liver [3,6]. The predicted *GFGΔ2/3 *isoform does not contain the epitopes recognized by either of these antisera as the splice event introduces shift in the open reading frame, upstream of the MutT domain [6]. In addition to the GFG isoforms shown here, the Celera rat genome assembly database also predicts 2 N-terminally truncated GFG isoforms consisting of the C-terminal 160 amino acids (protein id = EDM01308.1) and C-terminal 147 amino acids (protein id = EDM01307.1) relative to the full length 35 kDa protein [20,21].

### GFG contains a Mitochondrial Targeting Signal Peptide

**Figure 2:**
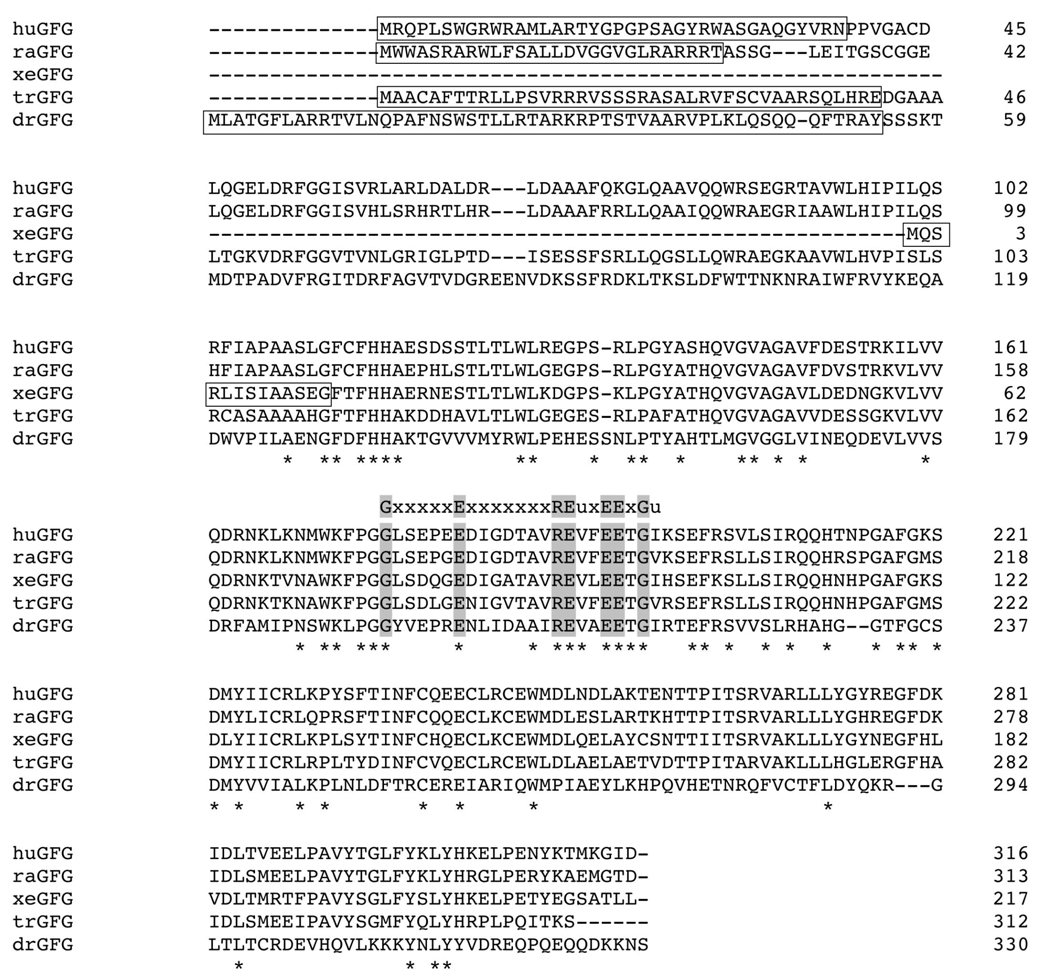
**Alignment analysis of GFG homologues**. Alignment of five GFG homologues was performed with ClustalW. Amino acid identity across all species is indicated by asterisks (*) below the sequence. The Nudix motif (GxxxxxExxxxxxxREuxEExGu) is indicated and amino acid identities within the motif are highlighted. The predicted mitochondrial targeting signal peptides (MTS) are boxed. The putative cleavage sites of mitochondrial signal peptidase are located after the last amino acid of the predicted MTSs. **Note**: hu = Homo *sapiens *(human); ra = *Rattus norvegicus *(rat); Tr = *Takifugu rubripes*; dr = *Drosophila melanogaster *(fruitfly).

**Figure 3:**
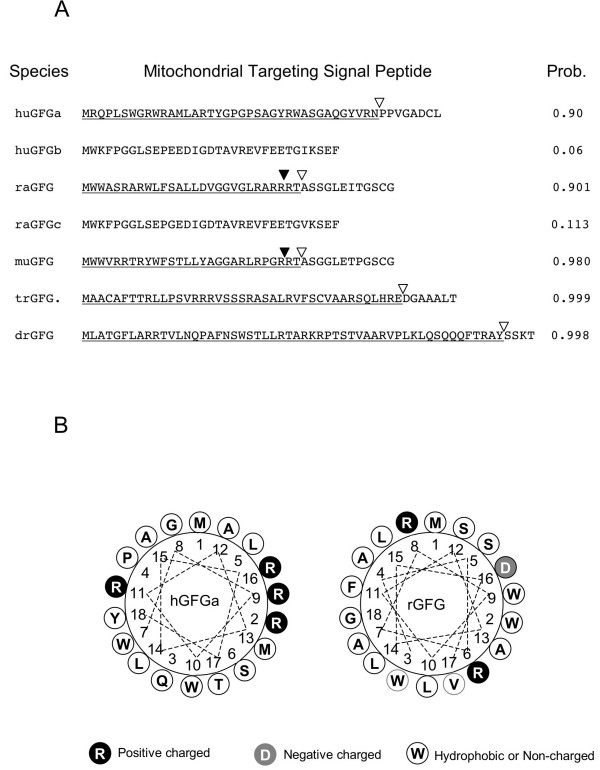
**GFG contains a predicted mitochondrial targeting signal peptide sequence**. (A) Mitochondrial targeting signal peptides were predicted in the N-terminal sequence of GFG from a several species using MitoProt II.0a The predicted signal peptide cleavage site is indicated by the open triangle. In rat and mouse, but not other species, a potential monobasic prohormone cleavage site was also identified (solid triangles). **Note**: raGFG: the N-terminal sequence is identical in raGFG, rGFGΔ2 and rGFGΔ2/3. raGFGc, an N-terminally truncated isoform predicted from the Celera rat genome assembly database, is >80 identical to human GFGb. **(B) **Edmunson helical wheel illustration of the human and rat MTS sequences. Hydrophobic and uncharged amino acids are depicted in white, positively charged residues in black, and negatively charged with gray circles.

The sequence of GFG is evolutionarily conserved from insects to humans particularly in the region of the nudix box domain (Fig 2). Although the sequences are more divergent at the N-terminus, comparison of twelve GFG homologues from a variety of animal species identified a potential mitochondrial targeting signal (MTS) sequence at the N-terminus in all species examined (Fig. 3a and Table 1). In contrast, GFG homologues from plants did not contain an identifiable N-terminal MTS (Table 1). Except for the MTS and the conserved nudix signature sequence, no other functional motifs or subcellular localization signal sequences were identified in any GFG homologues (not shown). TargetP and iPSORT identified an N-terminal signal peptide or subcellular compartment targeting presequence. In human only the hGFGa isoform contains an MTS sequence [16]. In contrast, in the rat exon 1 is common to all three previously characterized FGF-AS splice variants, and all three rat GFG isoforms contain a putative MTS (Fig 3a). However, the Celera rat genome assembly database also predicts an N-terminally truncated rGFG lacking the MTS sequence (Fig 3a, raGFGc). This predicted isoform shares more than 80% amino acid identity with human GFGb (NP_932158), which we have previously shown to be localized to cytoplasm and nuclei [16].

**Table 1:** Prediction of Mitochondrial Localization

***Protein***	**Program**			
	IPSORT (%)	LOCtree	MitoProtII.0a4	TargetPv1.01
	
hGFGa	yes	no	yes(0.872)	yes(0.864/28)
hGFGb	yes	no	yes(0.306)	no
hGFGc	yes	yes	no	no
rGFG	yes	no	yes(0.901)	yes(0.820/28)
rGFGΔ2	yes	no	yes(0.523)	yes(0.805/28)
rGFGΔ2/3	yes	no	yes(0.602)	yes(0.776/28)
rGFGc	no	no	no	no
murGFG	yes	no	yes(0.980)	yes(0.877/28)
droGFG	yes	yes	yes(0.997)	yes(0.832/32)
chiGFG	yes	yes	yes(0.321)	yes(0.352/20)
xenGFG	no	no	yes(0.334/13)	no
fugGFG	yes	yes	yes(0.999)	yes(0.956/25)
panGFG	yes	yes	yes(0.878)	yes(0.870/28)
ponGFG	yes	yes	yes(0.892)	yes(0.910/28)
dogGFG	yes	yes	yes(0.977)	yes(0.862/28)
mosGFG	yes	yes	yes(0.959)	yes(0.794/28)
atGFG	no	no	no	no
ceNudix1	no	no	no	no
hNUDT9	yes	no	yes(0.478)	yes(0.688/46)
hMTH	yes	yes	yes(0.639)	yes(0.644/34)
hMTY	no	yes	yes(0.501)	yes(0.612/14)

h = human; pan = *Pan troglodytes*; pon = *Pongo pygmaeus*. r = *Rattus norvegicus *(rat); mur = *Mus musculus *(mouse); dog = *Canis familiaris*; chi = *Gallus gallus *(chicken); xen = *Xenopus laevis*; Tr = *Takifugu rubripes*; dro = *Drosophila melanogaster *(fruitfly); At = *Arabidopsis thaliana*. mos = *Anopheles gambiae *(malaria mosquito); ce = *Caenorhabditis elegans*. hGFGa, -b and c represent human GFG isoforms; rGFG, -GFGΔ2,-GFGΔ2/3, and -GFGc indicate known and predicted rat GFG isoforms. yes/no = mitochondrial targeting signal peptide at the N-terminal of the protein has/not been identified; the numbers (ranging from 0.0 to 0.999) in the brackets indicate the probabilities of the predictions. The numbers behind the slashes indicate the predicted cleavage sites of the mitochondrial peptidases.

Although the N-terminal amino acid sequence of the full length rGFG differs from that of hGFGa, it is structurally similar, with a high content of positively charged residues, (21% in the MTS *vs. *12% on average) as well as hydrophobic and hydroxylated amino-acid residues. Furthermore, the N-terminus contains a potential mitochondrial processing peptidase cleavage site between residues 28 and 29 (Fig. 3a). Secondary structure prediction with ANTHEPROT shows that amino acids 3–17 of rGFG (and residues of 7–18 of the hGFGa) can potentially form an amphiphilic α-helix (Fig. 3B). These features are common to several well-characterized mitochondrial translocation signals [22,23]. Unlike in human, the N-terminal sequence of rat and mouse GFG also contains a monobasic prohormone cleavage site at arginine 26 (probability 0.99; NeuroPred [24]). Cleavage at this site eliminates the MTS, raising the possibility that alternative postranslational processing may also play a role in determining the subcellular distribution of rat GFG.

### Expression of GFG isoforms in rat tissues

**Figure 4:**
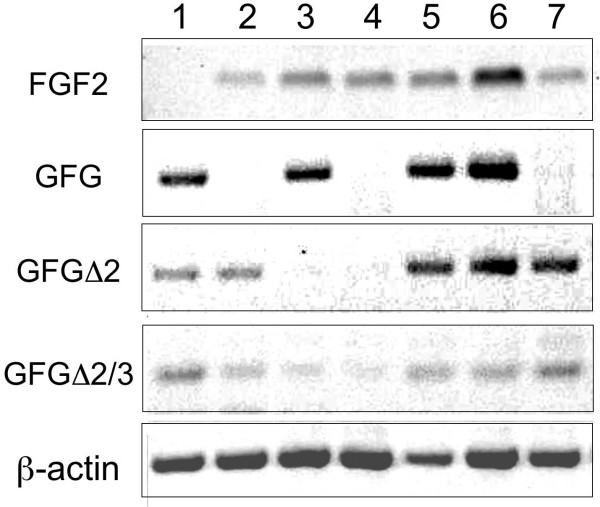
**Tissue specific expression of FGF2 and FGF-AS RNA in rat tissues**. Expression of FGF-2 and alternatively spliced FGF-AS mRNA isoforms was detected by PCR of a rat multitissue cDNA panel as described in Materials and Methods. Expression of β-actin was used as an internal reference. Lanes 1, liver; 2, brain; 3, spleen; 4, lung; 5, kidney; 6, skeletal muscle; 7, heart.

Expression of the FGF-2 and FGF-AS and their encoded proteins was examined in normal rat tissues. PCR of FGF-AS was performed using isoform-specific primer pairs to selectively amplify each of the major FGF-AS splice variants from commercially available rat multi-tissue cDNA panels. As shown in Fig. 4, GFG mRNA transcripts were detected in all tissues examined, although tissue-specific differences in isoform expression were observed. Only liver, kidney and skeletal muscle expressed all three FGF-AS isoforms at readily detectable levels. In contrast, in lung all transcripts of FGF-AS mRNA were at the limit of detection. The full-length transcript was abundantly expressed in liver, spleen, kidney and skeletal muscle, but not in heart or brain, which exclusively expressed the alternatively spliced transcripts. FGF-2 was detected in all tissues examined except liver. There was no obvious association between the level of FGF-2 and FGF-AS mRNA abundance in the tissues examined.

**Figure 5:**
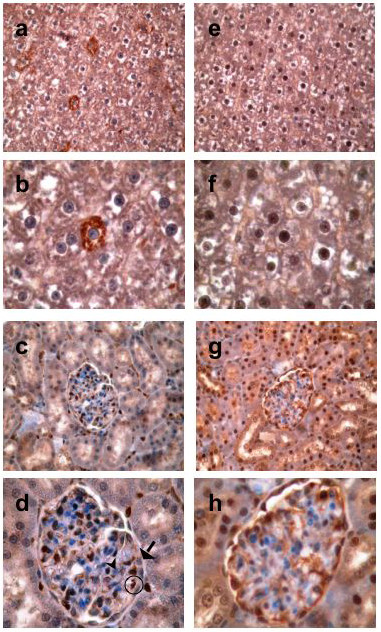
**FGF-2 and GFG protein expression in rat tissues**. Representative immunohistochemical staining of FGF-2 (panels a-d) and GFG (panels e-h) in rat liver (upper four panels) and kidney (lower four panels). FGF-2 immunoreactivity in liver was exclusively cytoplasmic (a, ×400; b, ×400 zoom). In contrast, GFG protein expression was detected in both cytoplasm and nuclei (e, ×400; f, ×400 zoom). In kidney, FGF2 staining is seen in glomerular endothelial and peritubular nuclei, but not in podocytes, mesangium or tubular nuclei. (c, ×400; d, ×400 zoom). In panel D, an FGF-positive endothelial cell nucleus is circled, and FGF-negative nuclei of a mesangial cell (arrowhead) and podocyte (arrow) are indicated for comparison. GFG immunostaining was detected in glomerular endothelial and peritubular capillary nuclei, and also in the nuclei and cytoplasm of podocytes and tubules (g, ×400; h, ×400 zoom).

Immunohistochemistry using a polyclonal antibody against the common C-terminus of GFG revealed that subcellular distribution of immunoreactivity was also tissue-specific (Table 2 and Fig. 5). GFG was strongly detected in both the cytoplasm and nuclei in many tissues, including liver, pancreas, kidney, testis, adrenal medulla, and in both small and large bowel. FGF-2 and GFG were co-localized in some, but not all tissues. In liver (Fig. 5, upper four panels), FGF-2 staining (Fig 5a, b) was strongly and exclusively cytoplasmic whereas GFG immunoreactivity (Fig. 5e, f) was detected weakly in cytoplasm and strongly in nucleus. In contrast, FGF-2 and GFG were co-localized in the nuclei of kidney glomerular endothelial cells and peritubular capillaries (Fig. 5c,d*vs. *5g,h). Consistent with our previous observations in human tissues, GFG was strongly expressed in the gastrointenstinal tract (Table 2). These observations suggest that subcellular localization of GFG is tissue-specific, and possibly isoform-dependent.

**Table 2:** Sub-cellular distribution of FGF and GFG immunoreactivity in rat tissues. Immunohistochemistry was performed as described in Materials and Methods. Tissues were scored as negative, -; a trace, (+); positive, +; or strongly positive, ++.

Tissue		Subcellular Distribution			
		
		FGF		GFG	
		
		Cyt	Nuc	Cyt	Nuc
Brain	cerebrum	(+)^a^	+^b^	+^a^	+^c^
	cerebellum	(+)	-	+	+
Spleen	lymphocytes	-	-	(+)	(+)
	Macrophages^d^	++	-	(+)	(+)
Lung	bronchial epithelium	(+)	+	(+)	+
	endothelium	-	+	-	+
	pneumocytes	-	-	-	-
Kidney	glomeruli	-	++ ^e^	+^f^	+ - ++^g^
	tubules	++	-	++	++
	peritubular capillaries	-	++	-	++
Pancreas	exocrine	++	-	++	++
	endocrine	+	-	++	++
Adrenal	cortex	-	-	-	-
	medulla	+	-	++	++
Uterus	endometrial glands	(+)	(+)	(+)	++
	endometrial stroma	-	-	-	(+)
	smooth muscle	-	-	-	(+)
Testes	germ cells	-	-	++	++
	Sertoli cells	-	-	++	++
	Leydig cells	++	++	++	++
Stomach	superficial crypts	-	-	-	-
	deep crypts	(+)	++	-	++
	smooth muscle	-	-	(+)	++
Ilium	glands	++	-	++	++
	ganglia	++	-	++	++
	smooth muscle	-	-	(+)	+
Colon	glands	++	-	++	++
	ganglia	++	-	++	++
	smooth muscle	-	-	(+)	+
Heart		-	-	(+)	+
Liver		+ +	-	+ +	+ +

^a ^white matter only; ^b ^oligodendrocytes; ^c ^oligodendrocytes and neurons; ^d ^sinusoidal macrophages; ^e ^glomerular endothelium only; ^f ^glomerular podocytes; ^g ^+ in glomerular podocytes, ++ in glomerular endothelium.

### Subcellular distribution of rat GFG

**Figure 6:**
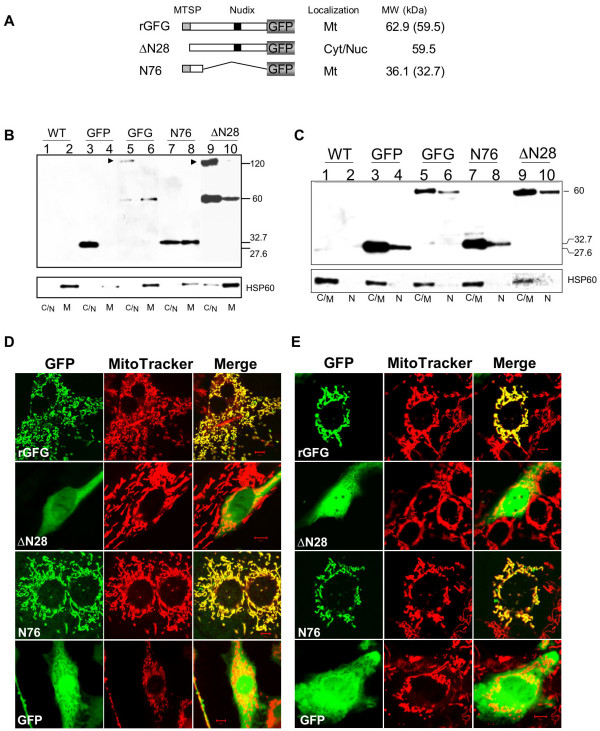
**Subcellular localization of rat GFG and deletion mutants**. **(A) Schematic representation of GFG:GFP fusion proteins**. The grey, black and hatched boxes represent the GFP, nudix and mitochondrial targeting signal peptide domains, respectively. rGFG indicates full-length rat GFG fused to GFP, ΔN28 indicates the GFG mutant lacking the N-terminal 28 amino acids encoding the predicted mitochondrial targeting peptide (MTS) of the rat full-length GFG. The N76 mutant encodes only the N-terminal 76 amino acids of GFG, including the MTS. The molecular weights and subcellular localizations are indicated at the right side of the diagram. Cyt//Nuc indicates that the fusion protein was localized in the cytoplasm and nucleus. Mt indicates the fusion protein was localized to mitochondria. The numbers in brackets indicate the predicted molecular weight of the fusion protein after cleavage of the N-terminal MTS. **(B, C) Subcellular fractionation and immunodetection of GFG:GFP fusions in stably transfected C6 cells**. Wild type (WT) C6 cells or cells stably transfected with the indicted GFP-GFG constructs were harvested and subjected to isolation of mitochondrial (M) vs. non-mitochondrial (cytoplasm + nuclei; C/N) fractions (**panel B**) or nuclear vs. non-nuclear (cytoplasm + mitochondria; C/M) fractions **(panel C)**. Following SDS-PAGE proteins were immunodetected with a monoclonal anti-GFP antibody or a polyclonal antibody to heat shock protein Hsp60 as a molecular marker of mitochondria in the fractions. Arrowheads indicate putative GFG dimers. **(D) Subcellular localization of GFG:GFP fusion proteins stably expressed in rat C6 glioma cells**. Stable transfectants were seeded in the wells of the chambered coverslips containing DMEM supplemented with 10% FBS and incubated overnight. Mitochondrial staining of stable cell lines was as described in Materials and Methods. The subcellular localization of the GFG:GFP fusions in the live cells was monitored with confocal laser scanning microscopy and a single slice of the stacks taken from a cell is shown here. The green channel shows the fluorescence of GFG:GFP fusion proteins (left panels), the red channel shows the fluorescence of the MitoTracker indicating the subcellular patterns of mitochondria (middle panels), the yellow fluorescence in the merged images (right panels) indicates the GFG fusion proteins targeted to mitochondria. **(E) Subcellular localization of GFG:GFP fusion proteins transiently expressed in rat C6 glioma cells**. Cells were transiently transfected with the indicated constructs. The images were taken as described in (D).

Although computational analysis predicts an N-terminal MTS in rat GFG, our previous results identified rat GFG immunoreactivity in the cytoplasm of transiently or stably transfected rat pituitary GH4 cells [18] and in the nucleus of rat C6 glioma cells [10,17]. To confirm the predicted N-terminal MTS experimentally, and to clarify these conflicting results, we examined the subcellular distribution of rat GFG using rGFG-GFP fusion constructs. The rat FGF-AS cDNA containing the complete open reading frame of GFG was fused in-frame to the 5'-end of green fluorescent protein (GFP) gene and inserted in a mammalian expression vector (Fig. 6A). The resulting plasmid DNA was first transiently transfected in the glioma C6 cells and its subcellular distribution pattern in live cells was monitored with confocal laser scanning microscopy 12–16 hours post transfection.

The green fluorescence of the fusion protein was exclusively detected in mitochondrial-like structures (data not shown and Fig. 6E, panel rGFG), while the green fluorescence of free GFP was evenly distributed in cytoplasm and nucleus (Fig. 6E, panel GFP). The mitochondrial localization of the rGFG-GFP fusion protein was further confirmed by dual monitoring of the red fluorescence of MitoTracker (which specifically stains mitochondria in live cells) and the green fluorescence of GFP. The yellow color in the merged images confirms that rGFG-GFP co-localizes with the MitoTracker dye in mitochondria. Expression of rGFG in stably transfected cells (Fig. 6D) gave the same results, indicating that the transfection method employed does not affect the subcellular distribution of GFG.

To confirm the integrity of the fusion protein and its subcellular targeting, a fractionation assay was conducted for immunodetection of the fusion protein in isolated mitochondrial (Fig. 6B) and nuclear (Fig. 6C) fractions. Immunodetection of HSP60 was used as a marker of mitochondrial enrichment (Fig. 6B, C; lower panels). No GFP immunoreactivity was detected in the wild type C6 cell, indicating the specificity of the GFP antibody (Fig. 6B, C; lanes 1 and 2). A ~60 kDa protein, the expected size of the GFG-GFP fusion protein, was predominantly identified in the mitochondrial fraction of transfected cells (Fig. 6B, lane 6), although a fainter band was also detected in cytoplasmic + nuclear (C/N) fraction (lane 5). The mitochondrial isolation kit used in this study yields highly purified mitochondria, but it does not remove all mitochondria from the remaining cytosolic + nuclear fraction, and it is likely that the immunoreactive band in the C/N fraction is of mitochondrial origin. This interpretation is supported by the confocal immunofluorescence data, which shows exclusive localization of GFG-GFP to the mitochondria. A 27 kDa protein, the expected size of free GFP, was exclusively detected in the C/N fraction (Fig. 6B, lane 3), but not in the mitochondrial fraction (lane 4) of the cells expressing GFP alone, confirming that mitochondrial targeting is GFG-specific. Subcellular fractionation into nuclear *vs*. cytoplasmic + mitochondrial (C/M) fractions gave similar results (Fig. 6C). rGFG-GFP was detected chiefly in the C/M fractions (Fig. 6C, lane 5). Although a small amount of GFG was detected in the nuclear fraction following subcellular fractionation (Fig 6C, lane 6) immunofluorescence was exclusively detected in mitochondria in intact cells (Fig 6D, E). High molecular weight bands detected for both the GFG-GFP and ΔN28GFG-GFP constructs are consistent with the molecular mass of possible GFG-GFP dimers (Fig 6B*arrowheads*) whereas free GFP (28 kDa) was detected only in its monomeric form. Dimerization of GFG would be consistent with the physiologically active form of many other nudix motif proteins [15,25,26], and we have recently demonstrated that human GFG exists in dimeric form in vivo [16].

### The N-terminal MTS is necessary and sufficient for mitochondrial localization

In order to confirm the role of the putative N-terminal mitochondrial targeting sequence, a GFG-GFP expression construct containing only the N-terminal 76 amino acids of rGFG was designed (N76; Fig. 6A). This construct was sufficient to target GFP to mitochondria in both stable and transient transfectants (Fig. 6C, D, panel N76), demonstrating that the C-terminal 228 residue region of rGFG is not required for mitochondrial targeting. In the Western blots, the ~32 kDa band detected in the mitochondrial fractions of the cells stably expressing N76 was smaller than the expected size (36 kDa), consistent with cleavage of the MTS by mitochondrial signal peptidase (Fig. 6B, C). The distribution of N76 fusion product in the cytoplasmic vs. mitochondrial fraction was increased slightly compared to the full length GFG-GFP construct (Fig. 6B, compare lanes 5 vs. 7). This may be due to decreased stability and consequent degradation of the deletion mutant protein during the protein isolation procedures. The confocal images confirm exclusive mitochondrial localization of the N76 deletion mutant in intact cells. A construct lacking the amino-terminal 28 amino acids of GFG (ΔN28) was detected predominantly in the cytoplasm and nucleus (Fig 6D, E). The integrity of the deletion mutant was confirmed with the Western blot assay (Fig. 6B, lane 9 and 10; Fig. 6C, lane 9 and 10). Some immunoreactive ΔN28 was detected in the mitochondrial fraction in western blots (Fig. 6B, lane 10) possibly reflecting a weak association of the fusion protein with the mitochondria. This is supported by the observation of the coincidence of the green fluorescence of the fusion protein with the red florescence of the MitoTracker in some transfected cells (Fig. 6E).

### Subcellular localization of alternative rat GFG isoforms

**Figure 7:**
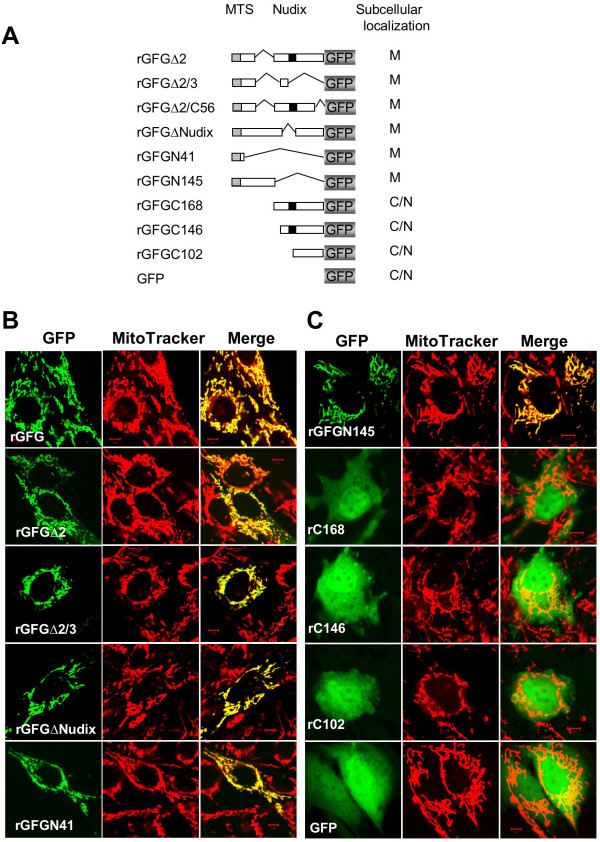
**Deletion analysis of rat GFG mitochondrial targeting**. **(A) Schematic representation of GFG:GFP fusion proteins**. The grey, black and hatched boxes represent the GFP, nudix and mitochondrial targeting signal peptide (MTS) domains, respectively. rGFGΔ2 indicates the rGFGΔ2 isoform in which amino acids (aa) 77–145 encoded by exon 2 are deleted by alternative splicing. rGFGΔ2/3, an alternative splice form lacking exons 2 and 3, encodes a GFG isoform containing only the N-terminal 76 aa of full-length GFG and a unique C-terminal tail consisting of 26 aa. rGFGΔ2/C56 is the GFP fusion of rGFGΔ2 mutant in which the C-terminal 56 aa were deleted. rGFGΔNudix is the GFP fusion of full length GFG from which the conserved nudix domain (23 aa) was deleted. rGFGN41, rGFGN145, rGFGC168, rGFGC146 and rGFGC102 indicate the GFP fusions of the N-terminal 41aa and 145aa, and C-terminal 168 aa, 146 aa and 102 aa of rat GFG, respectively. Their subcellular localizations are summarized at the right side of the diagram. C/N indicates the fusion protein localized in the cytoplasm and nucleus. M indicates the fusion protein targeted to mitochondria. **(B) **and **(C) Subcellular localization of GFG:GFP fusion proteins transiently expressed in the rat C6 glioma cells**. C6 cells were seeded in the wells of the chambered coverslips containing DMEM supplemented with 10% FBS and incubated overnight, then transfected with the plasmids expressing the indicated GFG:GFP. The subcellular localization of the GFG:GFP fusions in the live cells was visualized with confocal laser scanning microscopy and a single slice of the stacks taken from a cell is shown here. The green channel shows the fluorescence of GFG:GFP fusion proteins (left panels), and the red channel shows the fluorescence of the MitoTracker indicating the subcellular patterns of mitochondria (middle panels), The yellow color in the merged images (right panels) indicates the GFG fusion proteins targeted to mitochondria.

In addition to the full-length rGFG, we have previously cloned and sequenced alternatively spliced transcripts encoding 28 kDa (rGFGΔ2) and ~12 kDa (rGFGΔ2/3) isoforms of rGFG [6]. The 28 kDa and 12 kDa rGFG isoforms contain the same MTS at their N-termini. GFG-GFP fusion constructs of these two splice variants were generated (Fig. 7A) and their subcellular trafficking was determined in transiently transfected C6 cells. Both rGFGΔ2 and rGFGΔ2/3 were exclusively targeted to mitochondria, as were the deletion mutants GFGΔ2/C56 (lacking the C-terminal 56 residues), rGFGΔNudix (lacking the nudix domain) and rGFGN145 (lacking C-terminal 168 aa). In contrast, three N-terminal deletion mutants rGFGC168, rGFGC146 and rGFGC102, were localized in the nuclei and cytoplasm, but not in mitochondria, indicating that the N-terminal MTS is the only functional mitochondrial targeting signal in rat GFG. The deletion mutants rGFGC168 and rGFGC146 are structurally similar to rGFG isoforms predicted from the Celera rat genome assembly database, consisting of the C-terminal 160 amino acids (protein id = EDM01308.1) and C-terminal 147 amino acids (protein id = EDM01307.1). The latter isoform has 82% identity with the human GFG isoform b (GenBank accession number NP_932158), which we have previously shown to be nuclear and cytoplasmic in localization [16].

## Discussion

In the present study we analyzed the expression and subcellular distribution of rGFG, the protein product of the rat FGF-AS gene. Our present analysis shows that FGF-AS mRNA expression is ubiquitous, although individual splice variants were differentially expressed in a tissue-specific manner. Full-length rGFG was expressed in a limited set of tissues, and was not detectable in brain, lung, or heart. rGFGΔ2 and rGFG Δ2/3 were more broadly expressed, and detectable in varing levels in all tissues examined. FGF-2 and FGF-AS mRNAs were co-expressed, and immunoreactivity of their protein products were co-localized in some but not all tissues examined. There was no obvious association between the levels of expression of the sense and antisense mRNAs or proteins in these tissues.

Unlike the human GFG isoforms, all three of the previously characterized rGFG isoforms contained mitochondrial targetting signal sequences, and were shown to be exclusively targetted to mitochondria. The divergent N-terminal amino acid sequences of rat and human GFG indicate that the physicochemical properties of the MTS, rather than the specific amino acid sequence, determines mitochondrial targeting [27]. The primary structures of the human and rat GFG targeting sequences predict amphiphilic helices consisting largely of hydrophobic residues. Following mitochondrial import, the MTS is generally cleaved by mitochondrial processing peptidases [28], and the sizes of GFG:GFP fusion mutants observed in Fig. 6 are consistent with MTS cleavage. These results also indicate that the GFP fusion did not affect the structural conformation of the MTS [29].

We previously reported that human hGFG isoforms b and c, which lack the N-terminal MTS, are localized in the nucleus and cytoplasm, consistent with the detection of GFG immunoreactivity in these compartments in various primary human tissues [16,19]. Our present results indicate that rGFG, and the Δ2 and Δ2/3 isoforms are exclusively targeted into mitochondria. However, immunohistochemistry and sub-cellular fractionation studies identified cytoplasmic and nuclear GFG immunoreactivity in rat tissues and cell lines [6,17,18]. This suggests that additional rat GFG isoform(s), corresponding to hGFGb and/or hGFGc, remain to be identified. Indeed, the Celera rat genome assembly predicts a GFG isoform (rGFGc) with > 80% amino acid identity to human GFGb, and lacking the N-terminal MTS sequence. In the present study we confirmed that a deletion mutant of rGFG (rGFGC146), corresponding to the predicted sequence of rGFGc, is indeed localized in cytoplasm and nuclei of transiently transfected cells (Fig. 7), consistent with the behaviour of the corresponding human homolog, hGFGb. Expression of rGFGc may account for previous reports of nuclear GFG immunoreactivity in some tissues. Alternatively, tissue-specific posttranslational modification of rGFG may result in removal or inactivation of the MTS [30]. The N-termini of rat and mouse GFG (but not those of other species) contain a consensus monobasic prohomone cleavage site (Fig. 3a). This raises the possibility that, in some tissues, post-translational processing by prohormone convertases may remove the MTS, resulting in cytoplasmic and nuclear localization of rGFG. Prohormone convertase cleavage has previously been reported to mediate subcellular sorting of VGF [31].

The physiological substrate of GFG has not yet been identified. Although a plant homolog of GFG has recently been reported to have activity against ADP-ribose and NADPH [32], preliminary enzymatic analysis of GFG has failed to identify any typical nudix-like pyrophosphatase activity against a variety of substrates nor any MutT-complementation activity with rat or human GFG isoforms [16]. In contrast, expression and translation of FGF-2 and GFG are tightly linked [9,19], and dysregulation of their expression is associated with poor clinical prognosis in esophageal adenocarcinoma [16,33]. We have recently demonstrated that overexpression of GFG suppresses FGF-2 and inhibits cell proliferation in rat and human tumor cell lines [9,16]. Furthermore, the inhibition of cell cycle progression is reversible by addition of exogenous FGF-2 indicating that the phenotypic effects of GFG are directly attributable to its effects on FGF-2 expression [16]. This interpretation is supported by our demonstration that a GFG deletion mutant lacking the enzymatic nudix domain is as effective as the full length protein in suppressing cell cycle progression [9].

## Conclusion

In summary, FGF antisense gene encodes a highly conserved nudix motif protein present in all multicellular organisms. Alternative splicing of the primary transcript in animals (but not plants) results in multiple isoforms of the protein differentially targeted to mitochondria, cytoplasm and nucleus. However, biochemical and functional assays suggest that the primary function of FGF-AS is as a regulatory RNA controlling FGF-2 expression. The high level of FGF-AS sequence conservation in animal sequences suggests that this gene has been conserved to maintain its unique role in the regulation of FGF-2 expression.

## Methods

### Plasmid Construction

Standard molecular cloning techniques were used as described elsewhere [34]. To construct rat rGFG:GFP fusions for expression in mammalian cells, the cDNA of the open reading frames coding for rat GFG isoforms rGFG, rGFGΔ2 and rGFGΔ2/3 were amplified by PCR using plasmid DNA templates prGFGfl, prGFGspl1 and prGFGspl2 [3,6] and Pfu DNA polymerase. The resulting PCR-amplified DNA fragments were precipitated, digested with *Kpn*I and *Bam*HI, then purified and ligated into the corresponding restriction sites of pEGFP-N1, resulting in the plasmid constructs prGFG, prGFGΔ2 and prGFGΔ2/3. For prGFGΔNudix, the plasmid DNA of prGFG was digested with *Afl*III and the gel-purified larger fragment was religated resulting in a in-frame deletion of the Nudix domain of rGFG as described before [17]. N-terminal and C-terminal deletion mutants of rat GFG:GFP were amplified by PCR using template plasmid prGFG, Pfu DNA polymerase, and appropriate primer pairs. The PCR-products were precipitated, digested with *Kpn*I and *Bam*HI and inserted into pEGFP-N1, resulting in the plasmids prGFGN41, prGFGN76, prGFGN145, prGFGΔN28, prGFGC168, prGFGC146 and prGFGC102.

### RNA expression analysis

Expression in rat tissues was evaluated using Rat Multiple Tissue cDNA panel I (Clontech, Mountain View, CA). Expression of FGF2, as well as the full length (fl) GFG, GFGΔ2, and GFGΔ2/3 isoforms of GFG was analyzed separately, with Rat β-actin as an internal control.

Primer sequences were as follows: FGF2 forward: 5'-GAGGAGTTGTGTCCATCAAG-3' and FGF2 reverse: 5'-GGCCTTCTGTCCAGGCCCCG-3' with a Ta of 60 C, and yielding a 230 bp fragment: GFG-fl forward: 5'-GCTCTTGCAGGCCGCCATTCAG-3'and GFG-fl reverse: 5'-AAATACGGCACCTGCAACCCCTA-3' with a Ta of 57 C, and yielding a 226 bp fragment; GFG-Δ2 forward: 5'-TCTTGCAGGGTGCCGTATTT-3' and GFG-Δ2 reverse: 5'-CGGCTGCAGGCGGCAGATCA-3' with a Ta of 57 C, and yielding a 260 bp fragment; GFG-Δ2/3 forward: 5'-TTGCAGGTTGAAAAACATGT-3' and GFG-Δ2/3 reverse: 5'-CGGCTGCAGGCGGCAGATCA-3' with a Ta of 53 C, and yielding a 202 bp fragment; and β-actin forward: 5'-TGGCCTTAGGGTTCAGAGGGG-3' and β-actin reverse: 5'-ATCGTGGGCCGCCCTAGGCA-3', with a Ta of 57 C, and yielded a 244 bp fragment.

PCR amplification was carried out with an initial denaturation at 95 C, and 39 cycles of 95 C for 1 min, the product-specific Ta for 1 min, and 72 C for 1 min, and final elongation at 72 C for 10 min. Reactions were carried out in a 10 μL reaction volume, and used 1× Reaction Buffer, 0.4 mM dNTPs, 2.0 mM MgCl_2 _(Fermentas, Burlington, ON) for all except GFG-fl, FGF2, and β-actin, which contained 1.5 mM MgCl_2_, 0.8 μM each primer (Invitrogen, Burlington, ON), and 1.0 Unit of Taq DNA Polymerase (Fermentas, Burlington, ON), and 1.2 ng cDNA.

PCR products were visualized on a 1.5% agarose gel stained with ethidium bromide.

All products were sequenced (Robarts Research Institute, London, ON) to verify that the amplified fragments were derived from the correct target sequence.

### Immunohistochemistry

A modified immunoperoxidase technique was used to study the cellular distribution of FGF-2 and GFG proteins in formalin-fixed, paraffin-embedded rat tissue sections (Imgenex Histoarray, BIO/CAN Scientific, Mississauga, ON). Rabbit polyclonal anti-FGF-2 (Ab-2) was from Oncogene Research Products, Boston, MA. The GFG antiserum, raised against the highly conserved carboxyl terminus of rGFG, was used at a final concentration of 20 μg/ml as previously described [19]. Controls, including known positive and negative staining tissues, and samples in which the primary antibody was omitted, were run in parallel with the test sections. FGF-2 and GFG immunoreactivity was evaluated noting subcellular distribution (nuclear or cytoplasmic) using a scoring system previously described [19,35,36]. Intensity of staining was graded as negative, -; a trace, (+); positive, +; or strongly positive, ++.

### Computational analyses

GFG homolog search with the known rat GFG cDNA sequences [3-5] was performed using the NCBI BLAST server [37]. Multiple protein sequence alignments and phylogenetic analysis of evolutionary relationships of GFG homologs were conducted with Clustal W, a general multiple sequence alignment program [38]. SMART [39,40], a "Simple Modular Architecture Research Tool", was used to look for functional domain and signaling peptides. Nuclear localization signals were analyzed with PredictNLS server [41]. N-terminal signal peptide predications were performed with iPSORT WWW Service [42], LOCtree [43], MITOPROT [44], and TargetP Server v1.01 [45,46] which are freely available online. Prohormone cleavage site prediction was performed using Neuropred [24], available on-line [47].

### Culture, transfection and treatment of mammalian cells

C6 glioma cells were maintained in DMEM (Invitrogen) supplemented with 100 μg/ml streptomycin and penicillin, and 10% fetal calf serum (Invitrogen) at 37°C in a humidified atmosphere with 5% CO_2_. To establish stable cell lines, transfection of plasmid DNAs was performed according to the manufacturer's instruction using Lipofectamine™ reagent (Invitrogen). Transformants were selected in medium containing 1 mg/ml G418 (Invitrogen). The potential positive colonies were examined with epifluorescence microscopy for detection of GFP positive colonies. The colonies were isolated with cloning cylinders (Scienceware) by trypsinizing single colonies and amplified for immunodetection and laser scanning confocal microscope (LSCM). Transient transfections were conducted using transfection reagents FuGENE 6 (Roche Applied Science) or GeneJuice™ (Novagen).

### Protein extraction and subcellular fractionation

For subcellular fractionation assays, wild type (WT) C6 cells or cells stably transfected with the GFP-GFG constructs were harvested and subjected to isolation of mitochondrial vs. non-mitochondrial (cytoplasm + nuclei) fractions using Mitochondria Isolation Kit according to the manufacture's introduction of isolation of mitochondria using reagent-based method or nuclear vs. non-nuclear (cytoplasm + mitochondria) fractions using NE-PER^® ^Nuclear and Cytoplasmic Extraction Reagents (Pierce Biotechnology, Rockford, IL) following the manufacture's introduction. The Halt™ Protease Inhibitor Cocktail was added into the mitochondrial or nuclear extraction buffers before starting. The isolated mitochondrial pellets were dissolved in 1 × SDS-PAGE loading buffer[48] and boiled for 5 minutes. The protein concentration of all protein extracts were measured using Coomassie Plus™ – The Better Bradford Assay Reagent (Pierce Biotechnology, Rockford, IL) and then the extracts were stored at -80°C until use.

### Protein immunoblotting

Protein extracts and subcellular fractions were subjected to SDS-PAGE using a NuPAGE^® ^Electrophoresis System and 4–12% NuPAGE^® ^Novex Bis-Tris-Gels (Invitrogen). Immunodetection of GFG:GFP fusion proteins was performed using a mouse monoclonal anti-GFP antibody (*A.V. *Monoclonal Antibody of BD Living Colors™ Antibodies, BD Biosciences). The HSP60 antibody was a goat affinity-purified and pre-absorbed polyclonal antibody raised against a peptide mapping at the amino terminus of HSP 60 of human origin (identical to the corresponding mouse sequence, Santa Cruz Biotechnology, Inc.). The rabbit polyclonal anti-GFG antibody raised against a peptide of the C-terminal rat GFG was described previously [3]. The secondary antibodies were a blotting grade affinity purified goat anti-mouse IgG (H + L) horseradish peroxidase conjugate (Bio-Rad laboratories), a bovine anti-goat IgG conjugated with horseradish peroxidase (Santa Cruz Biotechnology, Inc.) and a donkey anti-rabbit IgG-Fab' conjugated with horseradish peroxidase (Amersham, plc). A nitrocellulose transfer membrane (Pierce Biotechnology, Rockford, IL) was used for immunoblotting. Immunoblots were visualized by ECL using a SuperSignal^® ^West Dura extended duration substrate kit (Pierce Biotechnology, Rockford, IL).

### Fluorescence Microscopy

Stably transfected C6 cells were seeded in a four-well collagen I-coated Lab-Tek^® ^II Chambered Coverglass (Nalge Nunc International) containing DMEM supplemented with 10% FBS and incubated overnight at 37°C in a humidified atmosphere with 5% CO_2_. For mitochondrial staining, the medium was changed to DMEM containing 100 nM MitoFluor Red 589 (MitoTracker, Molecular Probes, Inc., Eugene, OR), incubated for 30 minutes, and then replaced with normal medium without MitoTracker. The subcellular localization of the GFG:GFP fusions in the live cells was monitored with a ZEISS LSM 510 META laser scanning confocal microscope (LSCM, Carl Zeiss AG, Germany). Fluorescent image stacks were taken from a cell and analyzed using the software supplied (3D for LSM, Carl Zeiss AG).

For analysis of the subcellular localization of the GFG:GFP fusion proteins transiently expressed in C6 cells were seeded in chambered cover-slips containing DMEM supplemented with 10% FBS and incubated overnight, then transfected with the plasmid DNAs using FuGENE 6 transfection reagent. Six hours after transfection, the medium was replaced with the normal growth medium DMEM and cultured overnight. Mitochondrion staining and LSCM analysis was similar to that described for mitochondrion staining of stably transfected cells.

## Authors' contributions

SCZ generated the GFG-GFP constructs and carried out the subcellular localization studies. KAM carried out the PCR analysis of rat tissue GFG mRNA. MBN and LG carried out the immunohistochemistry studies. AGC and PRM participated in the design of the study. PRM conceived of the study, participated in its design and coordination and drafted the manuscript. All authors read and approved the final manuscript.
